# “I Just Have to Go and Heal”: A Qualitative Study on the Acceptability of the Belgian Sexual Assault Care Centres for Victims of Recent Sexual Assault

**DOI:** 10.3390/healthcare14091133

**Published:** 2026-04-23

**Authors:** Saar Baert, Mariska Meersschaut, Kristien Roelens, Sara Van Belle, Paul Gemmel, Iva Bicanic, Ines Keygnaert

**Affiliations:** 1 International Centre for Reproductive Health, Department of Public Health and Primary Care, Ghent University, 9000 Ghent, Belgium; 2 Department of Human Structure and Repair, Ghent University Hospital, Ghent University, 9000 Ghent, Belgium; 3 Institute of Tropical Medicine Antwerp, 2000 Antwerp, Belgium; 4 Department of Marketing, Innovation and Organisation, Faculty of Economics and Business Administration, Ghent University, 9000 Ghent, Belgium; 5 National Psychotrauma Centre for Children and Youth, University Medical Centre Utrecht, 3584 CX Utrecht, The Netherlands

**Keywords:** sexual assault centre, sexual assault, rape, qualitative methods, service delivery, service evaluation, acceptability

## Abstract

**Highlights:**

**What are the main findings?**

- Victims and support persons considered the Belgian Sexual Assault Care Centres’ model (SACC)—providing integrated medical and psychological care, forensic examination and the option to report to the police for victims of recent sexual assault—to be highly acceptable, valuing its holistic, accessible, and long-term approach to post-assault care.
- Key innovative components—embedded psychological support, forensic examination without mandatory reporting, and the option to report to police within the SACC—were viewed as beneficial.

**What are the implications of the main findings?**

- Strengthening coordination between SACCs and other criminal justice actors is needed to ensure continuity of support after acute care.
- Core features of the SACC model may inform the development or refinement of specialised sexual assault services in other countries.

**Abstract:**

**Background:** Sexual Assault Care Centres (SACCs) in Belgium provide integrated medical and psychological care, a forensic examination and the option to report to the police to victims of sexual assault (SA). Understanding victims’ acceptability of these services is essential for improving SACC’s effectiveness and informing policy. **Methods:** In-depth interviews were conducted with 19 victims and 14 support persons to explore victims’ experiences with SACCs. The victims represented diverse characteristics (gender, age, SACC site and police reporting status). Data were analysed using thematic framework analysis, guided by Sekhon’s “Theoretical Framework of Acceptability”. **Results:** Participants viewed SACCs as a highly acceptable integrated model of specialised care for victims of recent SA. They expressed strong appreciation for the care provided at the SACC and its set-up (affective attitude), and they identified key professional qualities of SACC professionals (ethicality). Participants demonstrated good understanding of the functioning of the SACCs (intervention coherence). The model was perceived as effective in providing medical care, mental health support, and facilitating police reporting, though gaps were noted in linking victims with other actors in the criminal justice system (perceived effectiveness). Organisational strengths included the holistic, long-term, proactive, affordable and accessible nature of the care offered (perceived effectiveness, burden and opportunity cost). Victims faced challenges in linking to, engaging with and remaining in care due to distress post-SA, with support persons playing a crucial role in helping them navigate these challenges (self-efficacy). **Conclusions:** The study highlights the acceptability of an integrated, multidisciplinary approach to specialised SA care. Key elements include embedded psychological support, the option for forensic examination without mandatory reporting, and the possibility of police reporting at the SACC. These findings may inform the development of specialised SA services in other settings.

## 1. Introduction

Sexual assault (SA) is a public health problem that affects individuals and societies across the globe [1]. Prevalence estimates indicate that sexual violence affects a substantial proportion of the population globally, with 27% of ever-partnered women aged 15–49 years experiencing physical and/or sexual intimate partner violence [2] and 6% experiencing non-partner sexual violence [3]. In Belgium, estimates based on behaviourally specific questions, which capture a wide range of hands-on forms of sexual violence, indicate that up to 42% of women and 19% of men aged 16 to 69 report lifetime experiences of SA [4].

The aftermath of SA often leaves survivors grappling with a range of physical, mental and reproductive health consequences [5,6,7]. These consequences may necessitate specialised healthcare to facilitate victims’ recovery. Reporting the SA to the police is also a victim’s critical first step for the case to progress into the criminal justice system. However, only few victims reach out for professional support post-SA, because they feel shame or embarrassment, have concerns around confidentiality, or are ambivalent towards care and support [8,9]. Those victims who do reach out for help often experience negative attitudes of healthcare workers or legal authorities [10].

To improve victims’ help-seeking experiences, lower the threshold for seeking care, as well as optimise the criminal justice process, specialised and comprehensive SA services have been increasingly established around the globe. They generally provide medical care and a forensic examination to victims of a recent SA, in combination with some form of mental health support and referral to follow-up services [11]. The perspective of victims is key to evaluate whether these services actually reach their objectives and improve victims’ health and well-being. So far, only a few studies have addressed victims’ perceptions on such specialised SA services [12,13,14,15,16,17,18]. The existing research from the United States (US) and the United Kingdom (UK) predominantly focusses on victims’ satisfaction with specific professionals involved with these services and the quality of care these professionals provide. Generally, victims are very positive about their experience with specialised SA services [12,13,14,15,16,17]. Often victim advocates, crisis workers and forensic nurses -being those who accompany and guide the victim throughout acute and follow-up care- are perceived more positively than professionals from the criminal justice system [13,14]. Victims identify the following behaviours and attitudes from the professionals as crucial: treating victims with respect and dignity, showing care and compassion, being non-judgmental, clearly explaining procedures, offering choices, believing and validating victims’ experiences, demonstrating cultural sensitivity, and being specialised and impartial [12,13,14,15,17,18,19]. Victims often perceive these professionals’ attitudes as humanising and facilitating their emotional recovery [16]. Additionally, victims also find the ease of access of specialised SA services while ensuring privacy, as well as its non-clinical environment, important [17,18].

**Figure 1:**
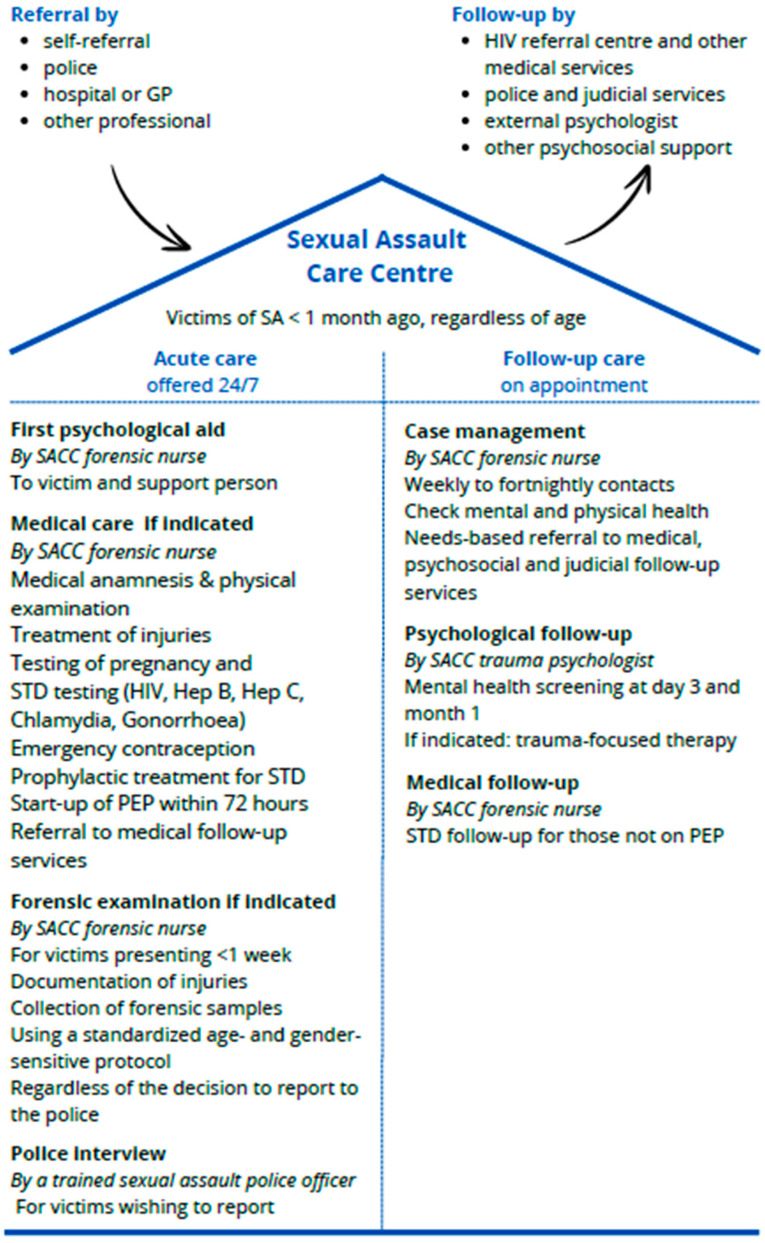
Model of the Belgian Sexual Assault Care Centres (SACC).

Since 2017, Sexual Assault Care Centres (SACC) have been established in Belgium. After a feasibility study [20,21], SACCs were piloted for one year in three cities. The history of its creation and the SACC model have been previously reported [22]. In summary (Figure 1), SACCs are one-stop centres located in a hospital that offer acute care for victims of recent (<1 month) SA, regardless of age, including first psychological aid, medical care, forensic examinations regardless of the decision to report to the police, and the possibility to report to the police with specialised SA police officers at the SACC after acute care. SACC follow-up services include referrals to medical follow-up services, such as the HIV reference centre (specialised hospital-based clinics providing post-exposure prophylaxis and HIV follow-up), as well as other psychosocial and judicial services, case management by phone, and support of an in-house trauma psychologist. Victims can self-refer, come accompanied by a police officer, or be referred by another professional.

SACCs are mainly visited by young female victims presenting for recent rape by a known assailant. Respectively 75% of victims received medical care, 61% a forensic examination, 50% psychological care, and 69% reported to the police [22].

The pilot of the SACCs aimed to test the intervention, allowing the programme designers to refine the intervention before a national scale-up. According to Klaic et al. [23], the scalability and sustainability of healthcare interventions is dependent on their acceptability for both users and providers, amongst other factors. The concept of acceptability has historically lacked a clear definition, despite its common use in process evaluations. The Theoretical Framework of Acceptability (TFA) was developed to guide quantitative and qualitative assessments before, during and after healthcare intervention engagement [24]. Acceptability is defined as a multi-faceted construct reflecting how appropriate individuals find a healthcare intervention, based on anticipated or experienced cognitive and emotional responses to the intervention. This theoretical framework includes seven constructs (Table 1): affective attitude, burden, ethicality, intervention coherence, opportunity cost, perceived effectiveness, and self-efficacy.

The present study aims to assess the acceptability of the Belgian SACCs for victims who attended these specialised services, with the prospect of improving the model. As the sparse previous research mostly used an interpersonal lens by assessing the satisfaction and the quality of the relationship with the professionals of specialised SA services, we aimed to broaden the understanding of clients’ experiences both on an interpersonal and organisational level by applying the TFA [24]. These insights are valuable not only for enhancing uptake and engagement with SACCs in Belgium, but also for other settings looking to implement or improve similar services.

## 2. Materials and Methods

### 2.1. Study Design and Setting

This qualitative study is part of a mixed-methods process evaluation of the Belgian SACC pilot [25]. The study was conducted in three SACC pilot sites: Ghent University Hospital, University Hospital Saint Pierre in Brussels, and Liège University Hospital.

### 2.2. Study Participants

Victims aged 16 or older who visited one of the three pilot sites between 25 October 2017 and 1 June 2019 were eligible to participate in the study. Support persons, such as family members or friends who accompanied victims to the SACC during that same period were also eligible to participate to broaden the range of collected victim experiences. Given that SA victims with posttraumatic stress disorder (PTSD) often avoid situations that remind them of the SA [26], which may result in disengagement from follow-up care [9], we anticipated that victims who disengaged from SACC follow-up care would also be reluctant to participate in an interview. By interviewing support persons, we aimed to include, at least indirectly, the experiences of those disengaging from care, as well as minor victims. Interviews with support persons were therefore treated both as proxy accounts of victims’ experiences and as reflections of support persons’ own interactions with the SACC.

Forensic nurses informed victims and their support persons about the study at the end of the acute care, and they asked for agreement to be contacted by a researcher for further information about an interview. Exclusion criteria included the inability to speak Dutch, French or English and the inability to provide informed consent. Out of the 1471 who attended one of the three SACCs during the study period, 543 consented to be contacted by a researcher. Consenting victims and support persons were then purposively selected by the researchers, applying principles of maximum variation and gradual selection [27], based on the following criteria: SACC site, gender, age and police reporting. The aim was to capture a diversity of experiences rather than to obtain a statistically representative sample of all victims attending the SACC. The researchers successfully contacted 92 individuals by phone six to nine months after their first presentation to the SACC enabling victims’ reflection on both acute and follow-up phases of care. The researchers explained the study aim and procedures and scheduled a time and place of the participants’ choice for those willing to participate. Of the 92 individuals contacted, 44 participated in an interview or focus group discussion. Some individuals declined participation (e.g., indicating they had moved on, found it emotionally difficult to revisit the experience or lacked time), while others disengaged during follow-up, either by no longer responding to contact or by not attending a scheduled interview after initial agreement. Participants were offered a €20 supermarket coupon as compensation for their time.

This study sample was restricted to participants in in-depth interviews (IDIs) concerning victims who attended the SACC within one month after the SA, as SACC services are primarily directed towards victims presenting within this time frame.

### 2.3. Data Collection

Semi-structured IDIs were performed between July 2018 and November 2019 by SB. Interviews were conducted in Dutch, French or English, at a location chosen by the participant. A topic guide (see Supplementary File S1) was developed by the research team based on the study objectives and informed by the existing literature on victims’ experiences with SA services and healthcare utilisation. The guide covered experiences with SACC services and other services intervening pre- or post-SACC, reasons for (not) reporting to the police, and (where applicable) the needs of support persons. Interviewers used probing questions to encourage reflection on both positive and negative experiences (e.g., “What was helpful?”, “What was less helpful?”, “What did you find inadequate?”, and “What aspects could be improved?”). In this study only data on the experiences with the SACC and other services were considered.

At the start of each interview, the study aims and informed consent procedures were re-iterated to the participants. Interviews were recorded using a voice recorder, and the interviewer also took additional notes. The audio recordings were transcribed verbatim by master’s students whose native language matched the interview language. Pseudonymised transcripts were checked for accuracy by SB, after which voice recordings were deleted. Data collection continued until thematic saturation was reached, defined as the point at which no new themes or relevant insights emerged from successive interviews [28]. Saturation was assessed iteratively during data collection and analysis, in line with the study’s purposive sampling strategy aimed at capturing a diverse range of experiences across key participant characteristics (e.g., site, age, gender, police reporting).

### 2.4. Data Analysis

Interviews were analysed using NVivo 14 using thematic content analysis [29]. First, SB, MM and IK familiarised themselves with the data, which informed the articulation of the research question and the selection of the dataset to be considered for this study. Second, SB, MM and IK developed an initial coding tree with themes deductively derived from the TFA [24] and the services offered at the SACC. Third, SB and MM applied this coding framework flexibly during an inductive coding process, allowing for additional codes to emerge that did not fully align with predefined constructs. These inductively derived codes were further organised into subcodes to capture variation and nuance within the data. Through this iterative process, the interpretation and delineation of TFA components were refined to better reflect participants’ accounts. All interviews were independently coded by SB and MM, while the first six interviews were compared to assess consistency in interpretation and align the coding strategy. Throughout the process, codes were collated into themes and subthemes, which were discussed by SB and MM to resolve discrepancies. Finally, SB refined and reorganised the themes in discussion with IK, KR, IB, SV and PG, shaping the overarching narrative of the analysis.

Support-person data were analysed alongside victim interviews without developing a separate coding structure; instead, patterns were compared across both groups to identify convergence and divergence in perspectives, allowing these accounts to complement and contextualise victim narratives.

Three authors (SB, KR and IK) were involved in the design, implementation and evaluation of the SACC model. While this proximity provided in-depth contextual knowledge of the intervention, it may also have introduced a risk of interpretive bias, including a tendency towards more favourable interpretations. Importantly, other members of the research team (MM, IB, SV and PG) were not involved in the implementation of the SACC model. To mitigate potential bias, data analysis was conducted collaboratively, with independent coding by SB and MM and iterative discussions to resolve discrepancies and refine interpretations. The development of themes was further discussed within the broader research team (SB, IK, KR, IB, SV and PG), allowing for critical reflection on emerging findings.

## 3. Results

### 3.1. Participant Characteristics

The qualitative sample comprised 33 victims, including 19 victims interviewed directly and 14 represented through support persons. Victim characteristics are presented in Table 2, which also provides an indicative comparison with the overall SACC population during the pilot year. The sample was comparable to the overall SACC population in terms of age and gender, while showing slightly higher service use and a different distribution across SACC sites.

Of the 14 adult support persons interviewed, 5 were male and 9 were female. The support persons included family members such as parents, partners or siblings (n = 10), professionals (n = 3), and other close contacts (n = 1).

### 3.2. Overview of Themes

Table 3 presents the main themes and subthemes identified in the data. The six main themes were derived from the TFA [24]: affective attitude, ethicality, burden and opportunity cost, intervention coherence, perceived effectiveness, and self-efficacy. Findings were largely consistent across interviews with victims and support persons, unless stated otherwise.

### 3.3. Affective Attitude

As defined in the TFA [24], this theme reflects the feelings of victims and support persons regarding the SACCs. Participants were highly positive about the SACC intervention itself and the professionals working there, particularly the nurses.

*It was an extraordinary help. Otherwise, you’d be completely lost.*

(Victim 18)

*I felt good here [at the SACC] despite the seriousness of it [the sexual violence] all.*

(Victim 7)

*The nurses were perfect!*

(Victim 19)

Many participants expressed that their motivation to be interviewed stemmed from their gratitude towards the SACC and its professionals, as well as a desire for the intervention to be scaled-up to ensure similar positive experiences for future SA victims.

*Honestly, I really found it [the SACC] very good. I was totally convinced, which is why I wanted to give my time [for this interview]. It was because of that, because I think it needs to continue and that it’s worth it.*

(Victim 2)

### 3.4. Ethicality

The TFA [24] defines ethicality as the extent to which the intervention has good fit with an individual’s value system. In this study we interpreted this theme as behaviours and attitudes of professionals working at the SAC, which were strongly appreciated by the participants.

#### 3.4.1. Being Empathic

Most participants expressed strong appreciation for the empathy shown by the nurses at the SACCs during acute care and identified it as one of the best aspects of the SACC. SACC nurses were described as human, non-dramatic, welcoming, sensitive to their needs, available and attending victims’ basic needs.

*People who are immediately there to listen […] and who come towards you and try their best to help you and make you feel somewhat comfortable after what happened. […] I dealt with people who were really very nice and understanding.* )

(Victim 5)

*Yes, they approached it [the forensic exam] in a very gentle way, and they also said: ‘I’m going to do this, is that okay?’. And there was constant checking to see if everything was alright, and then again afterwards they asked if it was okay or not. So yes, they do take into account that it’s actually something unpleasant.*

(Victim 15)

While empathy was also reported in relation to SACC psychologists, it was especially the empathy of SACC nurses that stood out to participants. Victims’ reports about interactions with specialised SA police officers at the SACC were mixed: while many perceived them as very respectful, some described their attitude as cold and distant.

*Yes, they [the police inspectors] were also very professional and calm, and yes, that was also very good, you know. Yes, and friendly, yeah.*

(Victim 13)

*I found it very, very cold, with very little empathy. […] I thought their attitude was… Well, like police officers, actually. It doesn’t surprise me. […] But then, he can’t be like: “How are you? Are you okay?” They can’t act like that either. I think that’s normal.*

(Victim 4)

The empathy of the SACC personnel stood sometimes in sharp contrast with the interaction with professionals that intervened pre- or post-presentation to the SACC. Those victims and support persons who presented first at the police station before coming to the SACC reported mixed experiences, as well as those in touch with judicial services.

*I had called the police, and yeah… what time was it? Half past ten [PM]. And he [the police officer] just bluntly told me, ‘You shouldn’t come with that now, come back tomorrow during the day.’ I said, ‘Are you serious? Is what happened not serious enough? Isn’t it important?’ ‘No,’ they said, ‘there’s no one here anymore’, and so on… And meanwhile, the police chief overheard the conversation, took over the phone and said, ‘Come here right away’.*

(Support person 9)

#### 3.4.2. Giving Clear Information

The clear information provided by the nurses, explaining each step of acute and follow-up care, was greatly appreciated.

*The explanation was really thorough. Before we started [the forensic exam]—I think they gave me a good half an hour- they explained everything to me: “There will be this, there will be that…” He explained that the samples would be kept for a certain period of time, um… that if I decided not to file a complaint, they couldn’t force me to. Well, everything….*

(Victim 19)

*We received a lot of little brochures like that. And it was good because, again, when they explain everything to us, it is impossible to remember it all. Especially since I hadn’t slept for hours, I was tired […], and with everything that had happened…*

(Support person 3)

Yet with regard to the follow-up by the criminal justice system upon reporting to the police, many participants emphasised the difference, as they felt a complete lack of information. For many, this was perceived as very difficult to cope with, while a minority was unconcerned and trusted the criminal justice system to do its job.

*The fact that they leave you in the dark about your own investigation. And, um, it would be appreciated, for example, to be kept informed about even the progress.*

(Victim 1)

#### 3.4.3. Offering Choices

Study participants indicated that being offered choices fostered a sense of control over the situation. This was often mentioned in the context of the forensic examination, as well as in decisions about reporting to the police, and accepting follow-up care.

*It’s not: ‘Boom! We’re doing this, we’re doing that [during acute care].’ … It’s: ‘If you want.’ One always has the right to say no, to say: ‘Stop.’ It’s not an obligation, there’s a lot of respect.*

(Victim 18)

*But she was already reassured, because the person I spoke to at the [SACC] centre told me that she would not be pressured to file a complaint, […] that she would be free [to decide what she wants].*

(Support person 11)

#### 3.4.4. Specialised Professionals

Many study participants felt relief and comfort knowing they would be attended by a service specialised in SA. Simply knowing this made it easier for victims to seek help, speak openly, and accept care.

*I think the care is precious, and completely different than when you would show up at the emergency department, with doctors who may be very nice but don’t necessarily do this all the time. And there [at the SACC], you really felt a willingness and a certain expertise from the nurse. She knew how to choose her words, she knew how to ask the difficult questions correctly as well.*

(Victim 17)

*Because the nurse told me that they are specialised police officers dealing with sexual offences who work exclusively with the [SACC] centre. And that it’s not like going to the police station.*

(Victim 8)

*The person told me that he [psychologist] was actually specialised in everything related to trauma. That’s when I understood that he wasn’t just a psychologist. Because it [SA] requires a different kind of knowledge, a specialisation.*

(Victim 7)

#### 3.4.5. Gender Sensitivity

Most victims were attended by female staff. Some victims specifically mentioned that being consulted by a female nurse, particularly for the forensic exam, or a female police officer was highly appreciated, as it made them feel more comfortable. Nevertheless, those seen by male personnel, commonly the SA police officers, did not necessarily view it as a negative experience.

*[It were] all women at the SACC. Yes, I think that’s also a good thing, after sexual violence. Because yeah, if a guy then comes to take care of you… I don’t think that’s going to work out. […] Yeah, I wouldn’t have wanted that. […] Especially when they do those tests in your intimate zones.*

(Victim 14)

*There was… a man and a woman [police officers]. And uh, I think the man had a daughter around my age. And he was a bit, uh… affected, and you could see it in his attitude. So he was very understanding with me, a bit protective, and uh… it’s more pleasant like that.*

(Victim 6)

### 3.5. Burden and Opportunity Cost

According to the TFA [24] burden refers to the perceived amount of effort required to participate in the SACC intervention, while opportunity cost concerns the extent to which benefits, profits and values must be given up to engage in the intervention. Although conceptually distinct, participants’ accounts in this study did not clearly differentiate between these domains. Given this empirical overlap, and the integrated nature of SACC services across acute and follow-up phases, these themes were analysed and reported jointly.

#### 3.5.1. Nearby Services

Most study participants mentioned that the physical distance to the SACC was manageable for acute care. For those who were referred by the police, this access was facilitated by police officers, which was perceived as convenient. However, some participants noted that even living nearby, they had to travel a long time to reach the SACC by public transport. Others expressed concerns about potential access issues once the SACCs would become widely known and attract victims living further away.

*[I drove her by car] because taking the train wasn’t really manageable for her. Especially since we had been clearly told over the phone that we needed to bring everything, like sheets, tissues, her clothes, and that she shouldn’t wash herself… […] I feel like it wasn’t ideal for her to deal with public transport because it was quite a long trip, and, it’s silly, but [she] doesn’t always have 4G to look at Google Maps. So, if she had had to go there alone, I’m not sure it would have happened either.*

(Support person 11)

Distance became more of a challenge for study participants regarding follow-up care with the SACC psychologist, leading some to seek a psychologist closer to home.

*And we’re also from [another city], so it’s always a thirty-minute drive to get here, which is also an obstacle to participating [in the psychological support].*

(Support person 9)

#### 3.5.2. Available Services

Participants perceived the acute SACC services as highly available. They appreciated not having to wait or book appointments, the SACCs 24/7 availability, and being attended to quickly upon arrival:

*She didn’t have to wait x amount of time, make an appointment, and then… It happened right away, you see. It’s important that it’s in the moment, not two months later when we can, uh… […] Yeah, it’s in the very moment that we need to immediately put words to it and see someone.*

(Support person 10)

However, when multiple victims were present, some noted experiencing long waits before being attended. Study participants also valued the availability of the case manager, who could be contacted at any time for support:

*In the evening, I had anxiety attacks. But here [at the SACC], we can call 24/7. So that helps too. So, when you don’t feel well all of a sudden… Hop, I can talk to someone.*

(Victim 8)

The availability of the SACC psychologist posed challenges for some participants. Some victims or support persons found it difficult to fit appointments around their regular activities, such as work or study, leading some to disengage from psychological care at the SACC.

*It was quite complicated to see a psychologist here at the SACC who would agree to schedule an appointment at 6 or 6:30 PM, after my work. […] Now I have made an appointment with another [psychologist. There] it’s open until 8 PM.*

(Victim 19)

Those who did not use or stopped follow-up services often acknowledged that these services were available to them at any time should they wish to re-engage with care.

*In the sense that I know, for example, if one day it comes back to me… a day when I cry, or a day when I’m not feeling well… I know I can call someone here [at SACC] to make another appointment with a psychologist, or to explain or to talk. […] And I think that is something really reassuring.*

(Victim 4)

#### 3.5.3. Lengthy Procedures

Many respondents described the long hours spent at the SACC, noting that this was challenging for both victims and support persons due to the intense emotions and fatigue.

*Actually, it’s really the time, and then all these emotions… You see, you’re assaulted, and then the police patrol comes to get you, you get treated here [at the SACC] where they ask you questions, you fill out documents, … And then you do the same thing with the police… And all of that takes hours and hours, and… […] I could think it would have been easier if we had done this or that. But in the end, I don’t really know, and I think that um…for the investigation and for a person’s health, it’s better to do everything as quickly as possible.*

(Victim 6)

#### 3.5.4. Affordable Services

Victims greatly appreciated that the SACC care was free of charge. For some, this was essential to seeking care, while for others, it held symbolic importance, as they felt they should not have to pay for the trauma they had endured.

*I’m a student, it’s not like I have a lot of… money to, um… you know. So that was also something that held me back from talking about it, because I thought, ‘Yeah, I’m not going to spend that much money on it,’ and then… then she [my friend] found [the SACC], so that… was a good option.*

(Victim 10)

*Personally… maybe it wouldn’t have been the reason that I would say “I’m not going”. But, I don’t know, it would make it seem a little bit more complicated, and in a way unfair. Because this [SA] is something that someone didn’t choose. So,… I think I don’t deserve to have to pay. You’re already paying with all kinds of pay and everything.*

(Victim 3)

### 3.6. Intervention Coherence

This theme addresses the extent to which victims understand the SACC model and its relationship to follow-up support provided by other stakeholders.

#### 3.6.1. Understanding of SACC Services

Although it was sometimes challenging for study participants to recall the acute care, most had a good understanding of the holistic care provided at the SACC and its objectives. Some study participants struggled to distinguish the medical care from the forensic examination, which was understandable given that both acts were integrated according to the SACCs SOPs.

*So yes, they took blood samples, and then they took, like, a sample, probably to check if I didn’t have any diseases, and well, I don’t really remember everything they did. Um. […] They did all of that [samples to find traces of the perpetrator] too.*

(Victim 1)

However, some study participants had unrealistic expectations about the forensic examination’s outcomes and analysis, assuming forensic samples are always analysed.

#### 3.6.2. Understanding of Services Intervening Post-SACC

Overall, study participants had a good understanding of the medical follow-up services offered at the HIV referrence centre. In contrast, the functioning of the criminal justice system was largely unclear to those who reported the SA to the police. Judicial procedures were perceived as complex, participants expressed uncertainty about appointing a lawyer, and judicial victim support services were not well known.

*After the SACC, I didn’t really know how… what I was supposed to do legally. Or if I had to do anything. […] On day three or so [after the SA], I was thinking, ‘Should I actually do something? Because I’ve now filed the complaint, can I follow up on it, can I… yeah, I don’t know?’*

(Victim 12)

### 3.7. Perceived Effectiveness

The TFA [24] defines perceived effectiveness as the extent to which the intervention is seen as likely to achieve its intended purpose. In our study, this theme represents, first, the ways in which participants believed the SACC had helped victims, and second, aspects of the SACC model considered effective in achieving its goals and what services might be missing.

#### 3.7.1. Perceived Outcomes

- Feeling recognised as a victim of SA:

Study participants described how the SACC intervention helped them feel like legitimate victims. The nurses’ acknowledgment of their experiences and the care they received were perceived as a validation. For some, this acknowledgement marked the beginning of their healing process, while for others it empowered them to take further steps, such as reporting to the police.

*I think at least it helped her realize that something had happened and that she was legitimate, too. Because, in a way, I think that if we hadn’t gone through the centre and there hadn’t been any samples taken, for example, I’m not sure she would have filed a complaint…*

(Support person 11)

*So, for the first time, I felt like I was in a legitimate victim position and no longer… Because [after the 1st SA for which victim did not go to a SACC] they made me feel like it was practically my fault and that I was bothering by asking for help.*

(Victim 7)

- Emotional support:

Study participants highly valued the emotional support and psychoeducation provided by the nurse during the acute care and throughout case management. Many considered their support one of the most important aspects of the SACC, helping them to progress and start their psychological recovery process after the SA. The psychologist was also perceived as a source of this support.

*They [the nurses] helped me a lot, voilà… It wasn’t a big deal, but they made me understand that it was normal if I wasn’t feeling well or if I had strange reactions, etc. They reassured me a lot and comforted me too, so…*

(Victim 7)

Those who lacked a support system were relieved that the SACC professionals provided a listening ear.

*Because I didn’t dare to talk about it with anyone else… and that there [with the nurse on the phone] I could… I could say that it didn’t go well… that something was wrong, or…*

(Victim 11)

- Dealing with trauma of the SA:

Several participants highlighted that support from the psychologist helped them manage acute stress symptoms and other psychological consequences of SA.

*I was actually treated there as well, you know. […] Yes, it [psychological support] pushed me to face it directly, and it has been tough months to process that. But otherwise, maybe it would have been buried for years.*

(Victim 15)

*I think it’s important for me to… to keep seeing [psychologist’s name] for now. […] Because I still am not sleeping, and all that, you know…. But at the same time, I’m now again able to leave the door open. […] So, I find that… I have to keep getting this help, because it’s really a step-by-step thing.*

(Victim 3)

Additionally, participants mentioned receiving support from the psychologist for other challenges, such as managing violent partners or navigating difficult relationships with parents.

- Reassurance about their health:

Several study participants reported feeling reassured about health concerns, including fears of HIV and other STIs, through the medical care provided at the SACC and the HIV referrence centre.

*Because it [the SACC] removes medical anxiety, clearly. So at least we feel calm about that. Even if we had picked up something, it might have been caught in time.*

(Victim 17)

- Facilitate access to the criminal justice system:

Several study participants noted that the SACC was effective in gathering evidence about the SA through forensic examinations, regardless of their decision to report to the police. Those undecided about reporting during acute care viewed this approach as a way to safeguard their options should they decide to report later.

*I think it’s good to do it [the forensic exam], it’s important to do it. And as they say… as I explained to you, it allows us to think about whether we want to start a [criminal justice] procedure or not.*

(Victim 5, did not report)

Study participants also believed that the SACC offered an effective means to report to the police in a timely manner and under suitable conditions by providing the possibility for an interview with a trained SA police officer at the SACC.

*Since I didn’t have to leave [the SACC], that they [SA police officers] were coming, that I had time to rest in the meantime… Yes, I think that played a role too. If you had told me, ‘You need to go to the police station to file a complaint,’ I would have said no. I would have gone home, I would have slept, and I wouldn’t have filed a complaint right away.*

(Victim 4)

#### 3.7.2. Effective Care Pathways

- Centralised holistic care:

Study participants greatly appreciated that all care, as well as a police interview, was provided in one location, especially at a moment where they often felt unable to think or act.

*You know, when they sit you and they tell you, ‘We have the police here and we have the psychologist, and there is us, the nurses, and you also get to see the doctor. And everything is covered. You just have to come.’… It just makes you feel like ‘OK, I just have to go and heal.’*

(Victim 3)

The provision of all care in one location ensured that their other needs—often not yet recognised as such—were also addressed, if they wished.

*They asked me [on the phone] what kind of support I wanted, what support I would need. So, at that time, it was just psychological. But they said, ‘Well, in any case, just come… the nurse will talk to you, and we’ll see what… what you need. Because we think you need more than just a psychological evaluation, like also a gynaecological examination… you need all’.*

(Victim 19)

Some victim-participants preferred receiving additional services at the SACC, such as follow-up appointments for HIV, the option to report to the police at the SACC post-acute care, access to victim support groups, and legal liaison services.

*You can only speak to the police at the clinic on the first day [of admission…] Like… It shouldn’t be just only for those who present within] the first week [after the SA], you know? Because I know there was a certain point [later on] I was thinking, ‘OK, I think I want to speak to the police now’. But then, I would start thinking ‘Oo, I have to go to the police station, and…’. So, it would stop me again.*

(Victim 3)

*Yes, I find that it’s missing… a legal liaison, let’s say, of what to do afterwards… Because we have medical support, psychological support, etc. But we are left on our own with all the legal procedures afterwards, on what we need to do to defend our rights…*

(Support person 1)

- Long-term follow-up:

Study participants strongly appreciated the fact that care extended beyond acute support and included case management and follow-up by the trauma psychologist.

*The fact that they really accompany us, even afterwards. You know, there are many places where it could be like that [during acute care]. And once we leave it’s over, they don’t get involved in our stories anymore. But here [at the SACC], no…they really get involved in our story and in our ordeal.*

(Victim 6)

However, many study participants expressed that they did not need these follow-up services. They were confident in their ability to cope on their own, or mentioned that other resources in their network, such as support from a friend or ongoing care from a mental health specialist external to the SACC, were sufficient.

*I saw the psychologist once, but pfff… I’m already seeing a psychiatrist for other things. And that’s it. I’m someone independent. I fight my battles on my own.*

(Victim 18)

- Proactive support:

Victims valued the SACC nurse’s proactive phone check-ins with victims, recognising that they might not have reached out on their own despite needing support. This was considered as important for continuing follow-up care.

*I bury my head in the sand, so I prefer not to say anything anymore, not to talk about it anymore… And so, if they [SACC nurses] hadn’t called me, I wouldn’t have called… Maybe I wouldn’t have even gone to get the other medications, uh… […] So it’s good that they do that.*

(Victim 6)

However, some victims found it difficult to respond to the phone calls, as there was rarely a suitable moment due to their job or the presence of others, or they simply wanted to continue their lives without being reminded of the SA.

*Well, I think there was, well, the timing wasn’t great… in the sense that it’s a bit complicated I think, uh… Well, especially when we haven’t talked to our surroundings etc. and well, we’re not always… even able to respond, to discuss these things…*

(Victim 5)

Participants appreciated that follow-up care was organised for them, with the SACC nurses prebooking appointments and sharing information whenever possible, particularly regarding follow-up at the HIV reference centre.

*You know which doctor you need to see. They immediately make an appointment for you, so it’s well followed up, really. […] Imagine if you had to do everything yourself after seeing the doctor, the gynaecologist, and all the tests… […] And if you have to repeat your story every time about what happened, with each doctor, you don’t feel like doing that, right? So now that’s basically all taken out of your hands.*

(Support person 15)

- Continuity of care:

Some victims disliked the changes in care personnel during acute care or case management, while others were indifferent. Victims felt particularly attached to the nurse who had provided acute support.

*But for me it was difficult, because you kept seeing different people instead of the ones who had cared for you [during acute care]. So…yeah, that was a bit irritating. Because you open up to one person, and you want to see them again, um […] But it wasn’t too bothersome or anything. The others were also very friendly and helpful, but yeah…*

(Victim 1)

Continuity of care with external services was not always well-established. Some victims were referred to external psychologists but were reluctant to contact them, while others returned to previously known mental health specialists without always disclosing their SA experiences.

*He gave me someone else’s number [a psychologist]. But I won’t contact them. Um, I don’t want to explain everything again. I don’t know if that person will be a good fit for me or not.*

(Victim 7)

- Safe space:

Participants perceived the SACC as a space providing both physical and emotional safety due to its separation from the rest of the hospital, its restricted access, a focus on victims’ privacy, the non-clinical atmosphere, and access to food and drinks.

*It’s almost like…. it’s out of this harsh reality. It’s almost like an escape, a place where you will feel much better. It’s worth it to like struggle to go.*

(Victim 3)

Therefore, stepping out of the safe SACC bubble to attend external follow-up services was difficult for some. As previously mentioned, these participants suggested including these services at the SACC. However, others preferred not to return for follow-up at the SACC, as it reminded them of the SA and its aftermath.

### 3.8. Self-Efficacy

According to the TFA [24], self-efficacy relates to individuals’ confidence in their ability to perform the behaviours required to participate in the intervention. Applied to this study, we report in this theme on victims’ feelings and emotions post-SA that facilitated or hindered their capacity to seek help and remain in care, and the role of support persons in this help-seeking process.

Study participants noted that victims did not always perceive their experience as SA, which is an important step before seeking formal help. Support persons often played a significant role in helping victims recognise that what occurred was SA.

*But in her mind, it wasn’t framed as rape or sexual assault. She found it “strange”. She knew that it wasn’t normal. But I believe the process and the time it would have taken her to reach that conclusion would have, in some way, worked against her.*

(Support person 11)

Victims’ psychological state also hindered their linkage to the SACC. Many victims did not seek formal help immediately, felt clueless about what to do, or failed to access dedicated services by themselves. In these cases, both formal and informal support persons were often crucial in recognising victims’ need for care, identifying specialised SA services, organising appointments at the SACC and accompanying the victim there.

*I was still in shock, and it was her [my friend] who clearly said to me, ‘Listen, I think you need to go. Don’t wait too long. I know it’s not easy, but I think you need to go quickly.’ So she acted, because she felt I wasn’t ready to do so yet, and that by the time I was ready, it might have been too late for medical care. So it was she who had heard about it, who did some research, and found out there was one [SACC] here and that it wasn’t too far.*

(Victim 17)

*At first, there was denial. So I didn’t want to acknowledge what had happened. And after that…after two days, I thought, ‘No, something did happen, you need to realise that and get the necessary care’.*

(Victim 19)

Feelings of shame, guilt, sadness, fear and disgust related to the SA, along with feeling overwhelmed and fatigued, also made it difficult for victims to engage with acute care at SACC, especially during the forensic examination and police interview.

*Because you are examined completely, and those are things that bring a lot of pain and shame. Because… since that [SA] happened, you feel a constant shame. And then you have to show it to someone. That is… that is basically the naked truth, literally and figuratively, that you lay yourself bare. That was very difficult.*

(Victim 16)

Acute stress symptoms, such as avoidance and fear, often prevented victims from engaging in follow-up care at the SACC or other referral services. Some said they preferred to move on, and not be confronted again with the SA, others felt that follow-up care lost priority amidst their daily activities. For some, this avoidance of follow-up care was present despite recognising its benefit for their health and wellbeing. This ambivalence led some victims to disengage after acute care or early in follow-up, while others shifted between engagement and disengagement.

*So at that moment [the first weeks after the SA] you think, ‘Yeah, I’m doing fine, it’s okay…’. Plus, you also get phone calls [from the SACC], and when you’re constantly confronted with that… It [not answering the calls] was maybe a bit of a way to avoid thinking about it for a while or…*

(Victim 15)

*But I actually didn’t go [to my first consultation with the SACC psychologist], because yeah… I don’t really like talking. […] But I’ve come to realise now [months later] that… I think… I long for a psychologist who is specialised in this, and I should really go there to be able to close that chapter.*

(Victim 1)

## 4. Discussion

### 4.1. Summary of Main Findings

This study analysed data from interviews with victims and their support persons who attended the Belgian SACCs to assess the acceptability of these services for victims using Sekhon’s TFA [24]. SACCs were found to be highly acceptable, as they were perceived as effective in providing medical and mental health support, forensic examination, and reporting to the police. Several key qualities of SACC personnel and SACC organisational strengths were identified as crucial in the provision of specialised SA services.

The theme *affective attitude* demonstrated participants’ highly positive perceptions of the SACC intervention and staff, especially the forensic nurses who served as their first point of contact. This aligns with previous studies conducted in the US and the UK among primarily female adult and adolescent SA victims, which report high levels of satisfaction with specialised services such as the US’s Sexual Assault Nurse Examiner (SANE) programmes [12,13,15,16] and UK’s Sexual Assault Referral Centres (SARCs) [14], particularly in relation to the compassionate and supportive care provided by nurses.

Within the theme of *ethicality*, several key qualities of SACC personnel were identified that may have shaped participants’ affective attitude towards the intervention, including empathy, clear communication, offering choice, and specialisation. These aspects have also been reported in previous studies among victims of SA in the US and the UK, including survey-based and qualitative studies in SANE programmes [12,13,15] and SARCs [14,17,18]. In addition, as a systematic review of victims’ experiences with healthcare responses to SA [19] support these findings. Victims who reported the SA to the police at the SACC generally described their interactions with the trained SA police interviewers as positive, in contrast to the often negative experiences with police reported in previous studies conducted in the US among SA victims [10,13]. However interactions with police officers at Belgian police stations often remained problematic, highlighting the added value of police officers sensitised to SA and trained in a trauma-sensitive approach.

SACCs were implemented with the aim of improving the physical and mental health of victims of SA and facilitating their access to the criminal justice system. Findings within the theme of *perceived effectiveness* suggest to what extent and through which organisational features these goals may be met. First, in terms of improving victims’ physical health, many reported receiving necessary medical care at the SACC and the HIV reference centre, which reassured them about their health. This is further supported by findings from a descriptive study of the Belgian SACC pilot, which reported a high uptake of both acute and follow-up medical services among those attending these centres [22].

Second, with regard to victims’ mental health, many described feeling validated and supported in their experiences as victims of SA. They reported receiving emotional support from SACC nurses, and assistance from the SACC psychologist in coping with the trauma. Such positively perceived interventions by first responders, as highlighted in systematic reviews on early interventions following SA [30] and on social reactions to interpersonal violence [31], may be associated with improved mental health outcomes and lower levels of PTSD following SA, particularly when interactions are perceived as supportive and of high quality. In contrast to many specialised SA services, which often rely on external referrals for mental health support, as reported in a systematic review of SA services across high-income settings [11], Belgian SACCs offer specialist in-house mental health support, which was used by approximately half of those attending these centres [22]. Our findings confirm earlier hypotheses from a quantitative study of the Belgian SACC pilot, which examined predictors of victims’ use of in-house psychological support [32], suggesting that offering this support as part of affordable, centralised, holistic, and long-term care may encourage engagement. At the same time, barriers such as limited availability and accessibility of the SACC psychologist, along with victims’ avoidance of care, may limit uptake.

Third, SACCs aim to facilitate access to the criminal justice system. Participants noted the convenience of having forensic samples collected regardless of their decision to report to the police, as well as the option of a police interview at the SACC conducted by a trained SA police inspector after receiving acute care. To our knowledge, this integrated care pathway is uncommon in other specialised SA services and may partially explain why nearly 70% of those attending Belgian SACCs report to the police [33]. However, this study also highlighted ongoing issues with victims’ interactions with the criminal justice system before and after SACC care, which are addressed in other settings, such as in the UK, through the involvement of support workers acting as liaisons with the criminal justice system [17].

Overall, participants described the SACC model as supportive of recovery and access to care and support; however, these findings reflect victims’ perceptions rather than measured outcomes, and further longitudinal research is needed to assess sustained health and justice-related impacts of SACCs on victims.

Other organisational strengths, identified within themes of *perceived effectiveness* and *burden and opportunity cost*, included a proactive approach; affordable, available, and accessible care; the creation of a safe space; and continuity of care. Similar priorities were identified in the Belgian feasibility study conducted prior to the implementation of SACCs [21], which highlighted the need for accessible and integrated care [21]. These aspects have also been reported in qualitative studies conducted in the Netherlands and the UK among victims of SA, which emphasise the importance of proactive support, continuity of care, and accessible services that respect victims’ privacy [9,17,18].

Within the theme of *intervention coherence*, participants demonstrated a good understanding of the services provided. However, expectations around forensic examinations emerged as a specific aspect requiring attention, one that, to our knowledge, has not been further explored.

In the theme of *self-efficacy*, this study also identified victims’ challenges in linking with, engaging in, and remaining in acute and follow-up care at the SACC due to distress following SA. Challenges in accessing SA services have also been reported by SA victims in the UK [18], while ambivalence towards post-SA care, and the pivotal role of victims’ support networks, have been previously recognised in qualitative research in the Netherlands [9].

### 4.2. Reflections on the Application of the TFA in Specialised SA Care

Applying the TFA in the context of integrated care for SA victims revealed several conceptual considerations. While the TFA has predominantly been applied in survey-based and quantitative research, our qualitative analysis highlighted important challenges in its application to in-depth interviews about integrated post-SA interventions. First, throughout our analysis, we observed considerable overlap between the components of the TFA, as participants’ accounts often reflected multiple constructs simultaneously. To address this, we made minor adaptations in the operationalisation of certain constructs to better fit the empirical data, which were clarified at the beginning of each Results subsection. In particular, ethicality—defined by Sekhon [24] as the extent to which the intervention aligns with an individual’s value system—was interpreted through participants’ accounts of the behaviours and attitudes of SACC professionals that they valued. In addition, burden and opportunity cost were analysed jointly due to their conceptual overlap in participants’ narratives. We further limited burden to practical aspects (e.g., time, cost, distance, and duration), while emotional and cognitive challenges were captured under self-efficacy. This distinction allowed us to better reflect the specific challenges faced by victims following SA.

Second, participants’ affective attitude towards the SACC appeared to be shaped by a combination of constructs, including ethicality, burden, opportunity cost, and perceived effectiveness. In this process, affective attitude appeared to function as a higher-order construct, reflecting the overall appraisal of the intervention and being influenced by these underlying dimensions. This suggests that the components of acceptability may be hierarchically structured, consistent with theoretical models of health service quality that conceptualise overall service satisfaction as being shaped by multiple underlying quality dimensions [34].

### 4.3. Study Limitations and Strengths

The study’s strengths include its relatively large sample size compared to other qualitative studies in this field [9,15,16,17,18]. These studies largely focused on female victims, whereas this study included both victims identifying as female and male, allowing for a broader range of perspectives. The study also captured a wide range of victims’ lived experiences with the SACC. In addition, including support persons allowed for the inclusion of potentially alternative viewpoints, particularly from victims who were unwilling to participate. However, support persons may not fully reflect victims’ perspectives and may introduce additional interpretation bias.

Several limitations should be considered. First, there is potential selection bias in participant recruitment. Although the aim was to inform all victims about the study, this depended on SACC professionals and was not systematically recorded. As a result, we cannot distinguish between victims who were not informed, those unable to consent, and those who declined participation. This may have influenced the composition of the study sample. In addition, it is possible that only those with positive experiences were willing to participate in an interview. While the inclusion of support persons may have partially mitigated this bias by capturing additional perspectives, the risk of selection bias remains.

Certain groups may be underrepresented in this study. Victims who did not speak Dutch, French, or English were not included, which may have limited the representation of (undocumented) migrant victims. These victims may face additional barriers in accessing SACC services, hence influencing their perception on the acceptability of the model.

This study does not include the perspectives of victims who did not attend the SACC. However, this is consistent with the study objective, which was to evaluate the SACC model among individuals who used the service during its pilot phase. At that stage, the focus was on understanding users’ experiences with a newly implemented model of care, rather than evaluating the broader SA care system.

In addition, the experiences of victims who disengaged from follow-up care may have been partially captured through interviews with support persons. As anticipated, victims who did not engage in follow-up care—particularly psychological care—were more often represented by a support person in the study (Table 2). While this allowed us to broaden the range of perspectives, these proxy accounts may not fully reflect victims’ own experiences and should be interpreted with caution. However, findings were largely consistent across victims and support persons.

Furthermore, although the topic guide included open-ended questions and probing prompts inviting both positive and negative experiences, relatively few critical reflections were reported. Some participants did mention areas for improvement, such as services that they deemed were missing or were burdensome, but overall feedback was predominantly positive. This may partly reflect social desirability bias, as participants may have found it difficult to express criticism about services from which they received support during a highly vulnerable period following a traumatic event. At the same time, the predominantly positive accounts likely also reflect genuinely positive experiences with the SACC model. The SACCs represent a relatively novel and integrated approach to care in Belgium, and several participants contrasted their experiences with previously fragmented or unavailable services. In this context, the perceived value of the services may be both authentic and amplified by the contrast with prior care gaps. Nevertheless, it remains possible that certain critical perspectives were underreported, and this should be considered when interpreting the findings.

Finally, participants’ accounts may have been influenced by the time elapsed between their SACC visit and the interview which may have shaped how participants recalled and interpreted their experiences.

### 4.4. Implications for Policy and Practice

The preliminary findings of the overall process evaluation of the SACC [25] had a direct impact on practice. SACCs were scaled up nationally following the pilot, with each province now hosting one SACC, and adapted SACC SOPs [35] have been implemented. These improved SOPs included a standard and proactive offer of judicial victim support services for SA victims, facilitated by the case manager and supported by requests from prosecutors. Additional enhancements involve the inclusion of liaison roles within the police to address victims’ questions and increased flexibility regarding interviews by specialised SA police inspectors for delayed reporters. Communication campaigns to promote the SACCs have also been launched, not only towards victims but also to their support persons.

This study also has implications for policy and practice beyond Belgium, potentially informing the evaluation and development of similar services in other countries. The study findings demonstrate that various components of care—such as in-house trauma support, forensic examination without the need to report to the police, and the option to report to the police at the SACC—are highly acceptable to victims and should be considered for integration in other contexts. The call for a trauma-informed approach within the criminal justice system also warrants attention.

### 4.5. Implications for Research

Research on victims’ experiences with specialised SA services has largely focussed on their satisfaction with these services, and the quality of their interactions with service providers. This study highlights additional dimensions that should be assessed when evaluating the acceptability of specialised SA services. A broader system-level perspective is needed, including victims who do not access specialised services to better understand barriers to care, unmet needs, and gaps in service provision across the SA care pathway. In addition, attention should be given to underrepresented populations, whose experiences of access and acceptability may differ. Finally, further work is needed to refine the application of the TFA in complex health interventions and trauma research, particularly regarding the interrelations between constructs and their operationalisation in qualitative research.

## 5. Conclusions

The SACCs were perceived as a highly acceptable means of providing care and support to victims of recent SA. A deeper understanding of victims’ experiences with such services may support policy makers and practitioners in developing effective interventions for victims at high risk of experiencing negative physical and mental health outcomes following SA.

## Figures and Tables

**Table 1:** Seven constructs of acceptability as defined in the Theoretical Framework of Acceptability [24].

Construct	Description
Affective attitude	How an individual feels about the intervention
Burden	The perceived amount of effort that is required to participate in the intervention
Ethicality	The extent to which the intervention has good fit with an individual’s value system
Intervention coherence	The extent to which the participant understands the intervention and how it works
Opportunity cost	The extent to which benefits, profits and values must be given up to engage in the intervention
Perceived effectiveness	The extent to which the intervention is perceived as likely to achieve its purpose
Self-efficacy	The participant’s confidence that they can perform the behaviours required to participate in the intervention

**Table 2:** Characteristics of victims included in the qualitative study (n = 33) compared with the SACC population during the pilot year (n = 931).

	Participated in Person n (%)	Represented by a Support Person n (%)	Total n (%)	SACC Population During the Pilot Year (n = 931) ^c^ (%)
Gender				
Male	1 (5)	2 (14)	3 (9)	88 (9)
Female	18 (95)	12 (86)	30 (91)	843 (91)
Age				
Minor	1 (5)	8 (57)	9 (27)	268 (29)
Adult	18 (95)	6 (43)	24 (73)	663 (71)
Service characteristics				
Medical care	15 (79)	12 (86)	27 (82)	695 (75)
Forensic exam	16 (84)	12 (86)	28 (85)	564 (61)
Reporting to police	12 (63)	13 (93)	25 (76)	640 (69)
Case management ^a^	18 (95)	13 (93)	31 (94)	772 (91)
Psychological care ^b^	16 (84)	8 (57)	24 (73)	426 (50)
SACC site				
Brussels	8 (42)	5 (36)	13 (39)	462 (50)
Ghent	8 (42)	7 (50)	15 (46)	250 (27)
Liège	3 (16)	2 (14)	5 (15)	219 (24)
Total	19	14	33	

^a^ victim having received at least one follow-up phone call from the forensic nurse; ^b^ victim having received at least one consultation with the SACC psychologist; ^c^ the SACC population includes the 931 victims who presented at the SACC during the pilot year (25 October 2017 to 31 October 2019) [18], representing a subset of the population from which participants to the qualitative study were recruited.

**Table 3:** Themes and subthemes identified in interviews with victims and support persons, organised according to the Theoretical Framework of Acceptability [24].

Main Theme	Subtheme	
Affective attitude		
Ethicality	Being empathic Giving clear information Offering choices Specialised professionals Gender sensitivity	
Burden and opportunity cost	Nearby services Available services Lengthy procedures Affordable services	
Intervention coherence	Understanding of SACC services Understanding of services intervening post-SACC	
Perceived effectiveness	Perceived outcomes Effective care pathways	Feeling recognised as a victim of SA Emotional support Dealing with the trauma of the SA Reassurance about their health Facilitate access to the criminal justice system Centralised holistic care Long-term follow-up Pro-active support Continuity of care Safe space
Self-efficacy		

## Data Availability

The research complies with the European General Data Protection Regulation. Due to the sensitive nature of the personal data collected for this study, the interview transcripts cannot be publicly shared. However, researchers who are interested in accessing specific de-identified portions of the data for secondary analysis may submit a reasonable request to the corresponding author. Access to such data will be subject to approval by the ethics committee that oversaw this study and will require a data-sharing agreement ensuring the protection of participant confidentiality and the use of the data in a manner consistent with the original consent provided by the participants.

## Supplementary Materials

The following supporting information can be downloaded at https://www.mdpi.com/article/10.3390/healthcare14091133/s1; File S1: Interview Guide for Victims and Support Persons—Evaluation of the Belgian Sexual Assault Care Centres.

## Abbreviations

The following abbreviations are used in this manuscript:

GP	General practitioner
Hep	Hepatitis
HIV	Human Immunodeficiency Virus
IDI	In-depth interview
PEP	Post-exposure prophylaxis
PTSD	Posttraumatic stress disorder
SA	Sexual assault
SACC	Sexual Assault Care Centre (BE)
SANE	Sexual Assault Nurse Examiner programme (US)
SARC	Sexual Assault Referral Centre (UK)
SOP	Standard Operating Procedure
STI	Sexually Transmitted Infections
TFA	Theoretical Framework of Acceptability
UK	United Kingdom
US	United States of America
