# Supplementary material for: “I Just Have to Go and Heal”: A Qualitative Study on the Acceptability of the Belgian Sexual Assault Care Centres for Victims of Recent Sexual Assault
Source: Healthcare (Basel). 2026 Apr 23;14(9):1133. doi: 10.3390/healthcare14091133 (PMC13164171; doi:10.3390/healthcare14091133)
Supplement: Supplementary file 1 [file healthcare-14-01133-s001.zip › healthcare-4181247-supplementary.pdf]

Additional file 1: Interview Guide for Victims and Support Persons – Evaluation of the Belgian Sexual Assault Care Centres

Introduction

Good day, my name is [Interviewer's Name]. Thank you for being here today and taking time to speak with me. As I explained on the phone, we are conducting a study to evaluate the care provided at Sexual Assault Care Centres (SACC). Your experiences and insights are invaluable to improving support services for victims of sexual violence and their support persons.

Before we begin with the interview, I'd like to ensure you understand the study, its process and your rights. All this information is detailed in this information sheet, which I will explain to you. If you agree to participate in the study, I will ask you to sign the informed consent form. (explain information sheet + informed consent form).

With your consent, this conversation will be recorded to help us accurately capture your feedback. These recordings will be pseudonymized and securely stored, and they will be deleted after transcription. Do you consent to have this interview recorded? (start recording)

Section 1: Care at SACC

1.1. A few months ago, you (or the victim) received care at the SACC. Can you share how you (or the victim) **came to seek care** at the SACC?

- What happened in the first moments after the sexual assault?
- How did you (or the victim) come to know about the SACC? (e.g., referral, website, word of mouth)
- Who supported you (or the victim) in this process of seeking help after the SA?
- What type of support were you (or the victim) seeking after the sexual assault?

1.2. Could you describe the **care** you (or the victim) received at the SACC? (probe about first psychological aid, medical care, forensic examination, police interview at SACC, case management and psychological care, care for support person)

- What aspects of the care provided did you (or the victim) find helpful?
- Were there any aspects of the care that you (or the victim) found less helpful or inadequate?
- Can you tell me why you found these aspects of care helpful /not helpful?
- What did you think about the organisation of this care?

1.3. Can you describe how you (or the victim) were (was) **treated** at the SACC? (probe about interaction with forensic nurse, psychologist, sexual assault police officer)

- What did you appreciate in the way they treated you?
- What did you appreciate less or was inadequate in the way they treated you?

1.5. How **accessible** was the SACC for you (or the victim) (probe about location, hours of operation, for both acute and follow-up care)

1.6. What was your experience with the SACC **environment**?

- How did you (or the victim) experience the atmosphere in the SACC?
- What do you (or the victim) think about the SACC infrastructure?

1.7. What do you (or the victim) think about the **cost** of services provided at SACC?

1.8 What are according to you the **two best things** of the SACC? What are the **two most important things to improve** at the SACC?

Section 2: Support Services Beyond SACC

2.1. Were you (or the victim) **referred** to other services from the SACC ? If yes, can you tell me which ones (e.g., HIV referral centre, centre for child abuse, GP, social services, victim support services,…)?

2.2. How would you (or the victim) evaluate the **referral process** to these services? (probe about way of referral, information sharing,…)

2.3. What were you (or the victim's) **experiences** with these other support services? (map per service mentioned)

2.4. How **effective** were these referred services in supporting you (or the victim)? (map per service mentioned)

Section 3: Interaction with Criminal Justice System

3.1. Did you (or the victim) **file a report** about the sexual assault with the police?

3.2 How did you (or the victim) **decide whether or not to report** the sexual assault to the police?

- What motivated you (or the victim) to report the SA to the police, or what discouraged you from doing so?

- What role did people from your (or the victims') support network such as parent, sibling, friend or partner play in your (or the victim's) decision to report to the police?
- What role did professionals such as your GP, professionals at the SACC, or a social worker play in your (or the victim's) decision to report to the police?

3.3. What was your (or the victim's) experience with the **interactions with the police** (for those who filed a report)

- at the police station
- during the police interview at the SACC
- during the investigation

3.4. How did you (or the victim) experience the way the other actors within the **criminal justice system** handled the case? (e.g. prosecutors, judges…)

- How did you (or the victim) feel about the time it took for the criminal justice system to address the case?
- How were you (or the victim) treated?
- How well were you (or the victim) kept informed about the progress of the case?
- How do you (or the victim) feel about the outcome of the case?

Section 4: Appreciation of the interview

4.1 We now came to the end of the interview. Thank you for sharing your experiences and insights. Your input will be instrumental in improving care and support for victims of sexual violence and their support persons. If you have any **further thoughts or suggestions**, feel free to share them now.

4.2 Thanks a lot for your time and willingness to talk to me today. How did you **feel about the interview**? (probe about feelings during the interview, and any need for further support)

Conclusion

Your feedback and that of other interviewees will be anonymously summarized in a report that we will share with the Belgian government. This will allow us to further refine the model of the Belgian Sexual Assault care Centres. If you wish so, we can share the evaluation report with you. I'd like to thank you again for your time and effort.
